# CoMB-Deep: Composite Deep Learning-Based Pipeline for Classifying Childhood Medulloblastoma and Its Classes

**DOI:** 10.3389/fninf.2021.663592

**Published:** 2021-05-28

**Authors:** Omneya Attallah

**Affiliations:** Department of Electronics and Communications Engineering, College of Engineering and Technology, Arab Academy for Science, Technology and Maritime Transport, Alexandria, Egypt

**Keywords:** childhood medulloblastoma, histopathology, computer-aided diagnosis, convolutional neural network, long short term memory

## Abstract

Childhood medulloblastoma (MB) is a threatening malignant tumor affecting children all over the globe. It is believed to be the foremost common pediatric brain tumor causing death. Early and accurate classification of childhood MB and its classes are of great importance to help doctors choose the suitable treatment and observation plan, avoid tumor progression, and lower death rates. The current gold standard for diagnosing MB is the histopathology of biopsy samples. However, manual analysis of such images is complicated, costly, time-consuming, and highly dependent on the expertise and skills of pathologists, which might cause inaccurate results. This study aims to introduce a reliable computer-assisted pipeline called CoMB-Deep to automatically classify MB and its classes with high accuracy from histopathological images. This key challenge of the study is the lack of childhood MB datasets, especially its four categories (defined by the WHO) and the inadequate related studies. All relevant works were based on either deep learning (DL) or textural analysis feature extractions. Also, such studies employed distinct features to accomplish the classification procedure. Besides, most of them only extracted spatial features. Nevertheless, CoMB-Deep blends the advantages of textural analysis feature extraction techniques and DL approaches. The CoMB-Deep consists of a composite of DL techniques. Initially, it extracts deep spatial features from 10 convolutional neural networks (CNNs). It then performs a feature fusion step using discrete wavelet transform (DWT), a texture analysis method capable of reducing the dimension of fused features. Next, the CoMB-Deep explores the best combination of fused features, enhancing the performance of the classification process using two search strategies. Afterward, it employs two feature selection techniques on the fused feature sets selected in the previous step. A bi-directional long-short term memory (Bi-LSTM) network; a DL-based approach that is utilized for the classification phase. CoMB-Deep maintains two classification categories: binary category for distinguishing between the abnormal and normal cases and multi-class category to identify the subclasses of MB. The results of the CoMB-Deep for both classification categories prove that it is reliable. The results also indicate that the feature sets selected using both search strategies have enhanced the performance of Bi-LSTM compared to individual spatial deep features. CoMB-Deep is compared to related studies to verify its competitiveness, and this comparison confirmed its robustness and outperformance. Hence, CoMB-Deep can help pathologists perform accurate diagnoses, reduce misdiagnosis risks that could occur with manual diagnosis, accelerate the classification procedure, and decrease diagnosis costs.

## Introduction

Childhood brain tumors are the most common cancerous tumors among children accounting for nearly 25% of all pediatric tumors (Pollack and Jakacki, 2011; Pollack et al., 2019). They are considered the second primary rationale of death among children under 15 (Ailion et al., 2017). Among pediatric brain tumors, childhood medulloblastoma (MB) is believed to be the leading cancerous brain tumor among children (Iv et al., 2019). MB accounts for around 15–20% of the central nervous system tumors that affect pediatrics worldwide (Arseni and Ciurea, 1981; Polednak and Flannery, 1995). It evolves within the cerebellum on the posterior area of the brain and progresses rapidly to other parts of the brain (Hovestadt et al., 2020). According to the classification of WHO, there are four MB classes (Pickles et al., 2018). Precise and early diagnosis of MB and its classes is crucial to decide the appropriate treatment and follow-up procedure. This procedure will correspondingly lead to higher survival rates (Davis et al., 1998; Furata et al., 1998), slower disease progression, and avoid acute side effects that could occur if not diagnosed and treated in early stages. These side effects would affect children's movements and synchronization and reduce their quality of life.

Amid imaging modalities, MRI is used in the diagnosis of pediatric brain tumors (Manias et al., 2017; Iqbal et al., 2018). Radiologists experience some challenges, especially when diagnosing pediatric brain tumors (Sachdeva et al., 2013; Ritzmann and Grundy, 2018), like childhood MB and classifying its four classes (Fan et al., 2019). The reason is that several MB subtypes cannot show apparent dissimilarities within the visual appearance of MRI scans (Fetit et al., 2018) which could lead to misdiagnosis (Zarinabad et al., 2018). Thus, other imaging techniques, such as histopathology can be more appropriate for diagnosing MB (Grist et al., 2020). Histopathological examination of biopsy samples is presently thought to be the gold standard to accurately diagnose MB and its classes (Szalontay and Khakoo, 2020). The four MB categories described by the WHO are the classic MB, nodular MB, desmoplastic MB, and anaplastic/large cell MB. Classifying these four classes is essential (Ellison, 2010); however, minimal studies have examined the four childhood MB classes (Dasgupta and Gupta, 2019). Analysis of histopathological images manually is challenging due to the complexity and similarity among childhood MB classes and the small cell size and shape, and the correlation and orientation discrepancy of the different MB tumor classes. Besides, manual analysis wastes time. It also depends mainly on the experience and skills of the diagnostician (Dong et al., 2020). Even expert pathologists could provide distinct diagnoses concerning the MB class (Zhang et al., 2019; Anwar et al., 2020). Another challenge in the manual analysis is the lack of pathologists, especially in the developed and developing countries (Robboy et al., 2013). These challenges have raised the essential need for automated computer-assisted-based pipelines to decrease the burden of the pathologists in classifying the classes of childhood MB and assisting them in achieving high accuracy rates of classification (Das et al., 2018a).

Recently, computer-assisted methods have been extensively used to resolve many related medical problems, and they successfully achieved superior classification results (Ragab et al., 2019; Yanase and Triantaphyllou, 2019; Fujita, 2020; Kumar et al., 2020). However, fewer research articles have been conducted to classify the four childhood MB classes from histopathological images *via* computer-assisted-based approaches because of the shortage of available datasets. The previously acquired datasets are private or publicly available for classifying anaplastic MB and non-anaplastic, which is a binary classification problem. Therefore, most of the previous related studies performed binary classification to differentiate between anaplastic MB and non-anaplastic. For example, in 2011, Lai et al. (2011) presented a pipeline based on Haar wavelets, Haralick Laws texture features, and random forest (RF) classifier and attained a 91% accuracy. Similarly, Galaro et al. (2011) proposed a computer-assisted system based on Haar and MR8 wavelets and achieved an accuracy of 80% using the k-nearest neighbor (k-NN) classifier. The following year, Cruz-Roa et al. (2012) examined the influence of the bag of features histograms method constructed from Haar wavelet transform and visual latent semantic feature extraction methods on the performance of the k-NN classifier. The authors found that the bag of features using Haar wavelet transform is better and achieved an accuracy of 87%. Later, Cruz-Roa et al. (2015) made a comparative study to compare the performance of several feature extraction methods, such as sparse auto-encoder, topographic independent component analysis (TICA), and 3-layered convolutional neural network (CNN). The uppermost accuracy attained was 97% using the TICA method. In the same year, Otálora et al. (2015) merged the TICA with wavelet transform and utilized a 2-layer CNN for classification, attaining an accuracy of 97%.

The previous techniques endured some limitations. Initially, they were all based on traditional feature extraction methods with several parameters to be chosen manually, which increased the time needed before classification. These conventional methods may also be prone to error and are not suitable for all classification problems as they depend on user experience and skill to select the appropriate technique and adjust its parameters (Hssayeni et al., 2017; Basaia et al., 2019). For example, in the gray level co-occurrence matrix (GLCM) texture-based method, choosing the appropriate distance, *d* is essential. It should be of sufficient size to involve all texture patterns and at the same time, maintain the regional nature of the spatial dependence (Humeau-Heurtier, 2019).

Another example is the local energy pattern feature extraction technique, where the features extracted using such a method are invariant to the imaging settings (Zhang et al., 2012). Some of the previous approaches also rely on the textural features alone, which might fail to describe different dataset patterns (Hira and Gillies, 2015; Babu et al., 2017). For example, when images are noisy, the GLCM method cannot extract significant features from images distinguishing among patterns (or classes) (Humeau-Heurtier, 2019). Moreover, almost all these methods depend only on a single form of feature extraction to construct their classification model. These methods did not examine the impact of fusing several feature extraction methods on the accuracy of classification. Also, they are all constructed using a small dataset compromising on only 10 images. Lastly, they all perform binary classification to differentiate between non-anaplastic and analyptic (binary classification problem). On the other hand, multi-class category is essential to figure out the subclasses of childhood MB and decide the appropriate treatment and observation strategy according to each class.

Multi-class classification of the four classes of childhood MB is much more complicated than binary classification. Few research articles have investigated this multi-class classification problem. To the best of our knowledge, the first and only research group that investigated the classification of the four MB subtypes from histopathological images using machine and deep learning (DL) techniques were studies by Das et al. (2018b) who presented a diagnostic framework to diagnose the subclasses of such pediatric tumor. The authors initially segmented images using k-means clustering. Afterward, the authors pulled out morphological and color features. They extracted textural features, including GLCM, histogram of oriented gradients (HOG), and Tamura, in addition to the local binary pattern (LBP) and gray level run matrix (GLRM). Next, they reduced the feature space using principal component analysis (PCA) and used them to construct a support vector machine (SVM) classifier. They attained an accuracy of 84.9%. Later in 2020, the same authors utilized the same features but used multivariate analysis of variance (MANOVA) as a feature reduction method. They found out that the MANOVA method increased the classification accuracy to 65.2%, which is greater than that of 56.5% without MANOVA. In the same year, Das et al. (2020b) decided that instead of using individual sets of features (Das et al., 2018b), they produced various groups of fused features to study the influence of feature fusion and selected the best mixture of fused feature sets. They employed PCA and SVM and achieved an accuracy of 96.7% utilizing the four combined features. Later, in the same year, Das et al. (2020c) used two pre-trained CNN, including AlexNet and VGG-16, with transfer learning. They first used a soft-max classifier for classification and reached an accuracy of 79.3 and 65.4% for AlexNet and VGG-16 CNNs, respectively. They extracted deep features from these networks and employed an SVM classifier for classification, achieving an accuracy of 93.21 and 93.38% for AlexNet and VGG-16 features, respectively. Afterward, Attallah (2021) introduced a framework based on the deep features of three CNNs combined with PCA and discrete cosine transform and classified using four machine learning classifiers.

These previous methods have shown several drawbacks that can be summarized as follows: the approaches by Das et al. (2018b, 2020a) utilized traditional individual feature extraction methods that depend on textural analysis, color, or morphological operations. Drawbacks of conventional methods are discussed earlier. The disadvantages of using color features are their vulnerability to light situations and occlusion (Afifi and Ashour, 2012; Park et al., 2012). The limitations of morphological features like shape features are their dependence on the human experience, and they might not produce efficient results when used alone for classification (Liu and Shi, 2011). Furthermore, the pipeline presented by Das et al. (2020b) examined the combination of textural-based features alone to perform classification. Das et al. (2020c) utilized only DL features to build the classification model. In other words, the authors employed only individual DL features for classifying the MB subtypes, where every one of them was of enormous feature dimension. The authors did not examine the influence of fusing numerous DL features extracted from different DL approaches to take advantage of different DL architectures. Additionally, Das et al. (2020c) employed only two CNNs separately. The training time achieved utilizing these DL methods is enormous. Finally, some of them did not attain superior accuracy and cannot be considered as reliable systems. A reliable system means that it has a sensitivity that exceeds 80%, a specificity that exceeds 95%, and a precision that exceeds 95% (Ellis, 2010; Colquhoun, 2014). So, these systems did not achieve the criterion to be a reliable system.

The originality of the study may be listed in the following four contributions. First, a reliable pipeline called CoMB-Deep is built to classify the four classes of childhood MB with high accuracy. Second, CoMB-Deep is based on a composite of DL methods that are merged. Third, it blends the advantages of textural-based feature extraction and DL approaches using three stages. Initially, 10 CNN DL methods are utilized to mine deep spatial features. These deep spatial features are fused using discrete wavelet transform (DWT), a textural analysis-based approach capable of reducing the dimension of DL features. Afterward, the best combination of fused features is searched using two strategies to choose the appropriate set of fused features that influence the classification performance. Next, CoMB-Deep employs the bidirectional long short-term memory (Bi-LSTM) DL method, which illustrates the temporal information of the data, and not the spatial information like the CNNs used in the literature. The fourth contribution is the further reduction of the feature space dimension after the process of DWT-based fusion using two feature selection methods. We want to highlight that one of the core difficulties in classifying the childhood MB classes is the obtainability of the dataset.

## Methods

### Dataset Acquisition

**Figure 1:**
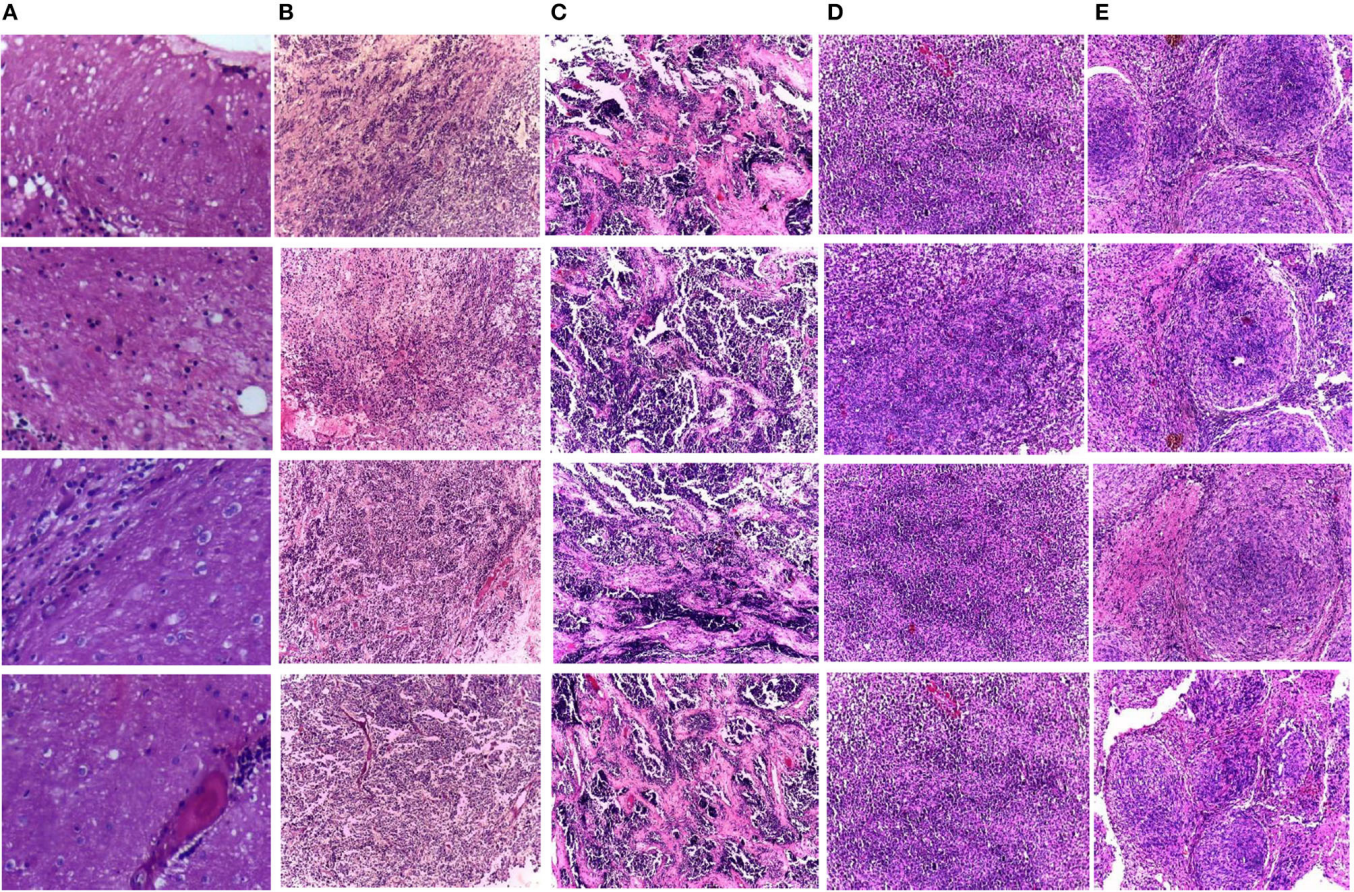
Samples of the childhood pediatric MB images, **(A)** normal, **(B)** classic, **(C)** desmoplastic, **(D)** large cell, **(E)** nodular.

Two medical centers for neurological research, including Guwahati Neurological Research Center (GNRC), and the Guwahati Medical College and Hospital (GMCH), involved collaborating medical institutes to acquire the dataset. The childhood MB dataset employed in CoMB-Deep was gathered from kids diagnosed with MB tumor. These kids were aged <15 years. Some information blocks were generated from those kids at the surgical department of the GMCH. The swabs were acquired through these tissue blocks. Next, Hematoxylin and eosin (HE) were employed to stain these sections of tissues at Ayursundra Pvt. Ltd., in which a local medical specialist supplied pathological help. Later, a certified specialist determined the region of interest for ground truth from such slide scans. Afterward, the images of these areas of interest taken with an amplification factor of 10x with a Leica 1CC50 HD microscope were stored in the Joint Photographic Experts Group (JPEG) format. The four subclasses of MB tumor were included in the dataset, which involved 204 images. Some of these images are displayed in Figure 1. The number of samples for normal, classic, desmoplastic, large cell, and nodule MB classes include 50, 59, 42, 30, and 23, respectively. Details of the dataset can be found in the study by Das et al. (2019).

### Methods of Deep Learning

During the last decade, a new branch of machine learning techniques called DL has been extensively used in many areas due to its high capability of overcoming the drawbacks of traditional artificial neural networks (Litjens et al., 2017; Attallah et al., 2020b). There are several architectures of neural networks based on DL. Among these architectures is the recurrent neural networks (RNNs), such as long-short term memory which are used for sequential data, and the CNN utilized for both image/video classification (Liu et al., 2017, Ceschin et al., 2018; Alom et al., 2019; Attallah et al., 2020c; Ragab and Attallah, 2020). The CNN demonstrates only spatial information from images; therefore, this type of DL technique is combined with Bi-LSTM, which determines the temporal data from the data given as its input.

#### Convolution Neural Network Architectures

The fundamental components of CNNs involve convolutional layers, rectified linear unit (Relu) layers, pooling layers, dropouts, fully connected (FC) layers, softmax and output layers (Alom et al., 2019). The organization of these layers may form a new architecture. The convolutional layer convolves a portion of the image with a filter of a small size. The outputs of the filter after passing through the whole picture are known as the feature map. The Relu layer applies the Relu activation function, which alters the entire negative activations to zero. It also enhances the non-linear characteristics of the CNN without influencing the receiving fields of the convolutional layer. The pooling layer is responsible for downsizing the massive dimension of the feature map of the previous layer. The most common types are maximum and average pooling. The dropout layer is used to prevent the network from overfitting by randomly adjusting the output edges of hidden neurons to zero at every training iteration. The FC layers are the last few layers of a CNN where the entire inputs of the previous layer are connected to each neuron in the subsequent layer. The softmax layer is responsible for the classification procedure using the softmax function, assigning probabilities for each class. The output layer produces the result of the network after training (Pouyanfar et al., 2018). Some of the state-of-the-art CNNs are briefly discussed below.

ResNet-50 CNN was proposed by He et al. (2016). The fundamental building block of ResNet is the residual block which involves shortcuts (known as residuals) within the layers of a traditional CNN to step over specific convolution layers at a time. This structure can enhance the performance of the CNN and accelerate the convergence process of the CNN, regardless of many deep convolution layers. ResNet-50 consists of 50 layers. The sizes and names of these layers are illustrated in Supplementary Table 1. In 2016, Google proposed the Inception-V3 CNN architecture (Szegedy et al., 2016b). It is based on the inception module that combines several convolutional filters of different capacities into a new filter. This process correspondingly lowered the number of parameters used in the training and the computational time. It consists of 48 layers, including convolutional, pooling, convolutional padded, and fully connected layers. The sizes and names of these layers are illustrated in the Supplementary Table 2. DenseNet was proposed by Huang et al. (2017). The main building block of DenseNet is the Dense block that connects every layer to every subsequent layer in the feed-forward procedure. At every layer, the feature maps of the earlier layers' are considered inputs to the current layer. DenseNet-201 consists of 201 layers. Its structure comprises a convolutional layer followed by a Dense block and a transition layer (Huang et al., 2017; Li et al., 2021). The transition layer consists of a convolutional layer followed by a pooling layer. The sizes and names of these layers are illustrated in Supplementary Table 3.

MobileNet is a lightweight CNN proposed by Howard et al. (2017). Its structure depends on two layers: depth-wise layers and point-wise layers. The depth-wise layer has numerous convolutional layers of 3 × 3 kernels. The point-wise layer consists of several convolutional layers of 1 × 1 kernels, where it contains 53 deep layers. The dimensions and names of these layers are illustrated in Supplementary Table 4. Inception-ResNet-V2 was proposed by Szegedy et al. (2016a), who inserted residual shortcuts in the Inception block of the Inception CNN structure. This network consists of 164 layers. The design of Inception-ResNet-V2 and the output size of layers are shown in the Supplementary Figure 1. Xception was introduced in 2017 by Chollet (2017). Its main building block is the Xception module. The Xception module switched the standard Inception modules with depth-wise separable convolution layers. This module begins with two convolutional layers, followed by depth-wise separable convolution layers, four convolution layers, and a fully connected layer without average pooling. The non-overlapping sections of the output channels from these layers are concatenated. Xception CNN has 36 convolutional layers.

NasNetMobile was introduced by Zoph et al. (2018). The main building blocks of NasNetMobile are called cell convolutional, which have a similar structure but different in weight. They are optimized using reinforcement. This network searches for the best architecture for a given dataset. ShuffleNet was proposed in 2018 by Zhang et al. (2018). Its crucial element is the ShuffleNet which delivers two new procedures known as point-wise group convolution and channel shuffle. The point-wise process utilizes a 1 × 1 convolution to decrease the computation time while attaining an adequate accuracy, whereas the channel shuffle procedure helps in streaming information among feature channels. It consists of 50 layers. The architecture of ShuffleNet is shown in the Supplementary Table 5. SqueezeNet was proposed by Iandola et al. (2016). The basic building block of SqueezeNet is the fire module. This module has a squeeze convolutional layer (having only a 1 × 1 filter), supplying an expand layer that consists of a mixture of 1 × 1 and 3 × 3 convolutional filters. The layers and their sizes are shown in the Supplementary Table 6. DarkNet-53 was initially proposed in 2017 by Redmon and Farhadi (2017). DarkNet-53 primarily consists of a sequence of convolution layers sizes of 1 × 1 and 3 × 3. It usually pairs with the number of channels after each pooling phase. DarkNet-53 has a total number of 53 layers (involving the last fully connected layer but not including the residual layer).

#### Bidirectional Long Short-Term Memory

The long short-term memory DL architecture (Hochreiter and Schmidhuber, 1997) was inspired by analyzing the stream of error in the RNN (Angeline et al., 1994). The LSTM architecture consists of an input gate, a forget gate, and an output gate. These gates are responsible for recognizing the long-range temporal reliance. The fundamental concept of the Bi-LSTM (Schuster and Paliwal, 1997; Baldi et al., 1999) is to introduce every training cycle forward and backward to two distinct LSTMs, both of which are associated with the same output layer.

### Proposed CoMB-Deep Pipeline

This study introduces a computer-assisted pipeline called CoMB-Deep to classify childhood MB subclasses from histopathological scans automatically. The CoMB-Deep consists of a combination of multiple DL methods to classify childhood MB and its subclasses. The classification is accomplished in two categories: binary and multi-class classification categories. The former category categorizes the microscopic scans into abnormal and normal (binary classification category). The latter category classifies the abnormal microscopic scans into four childhood MB tumors (multi-classification category).

**Figure 2:**
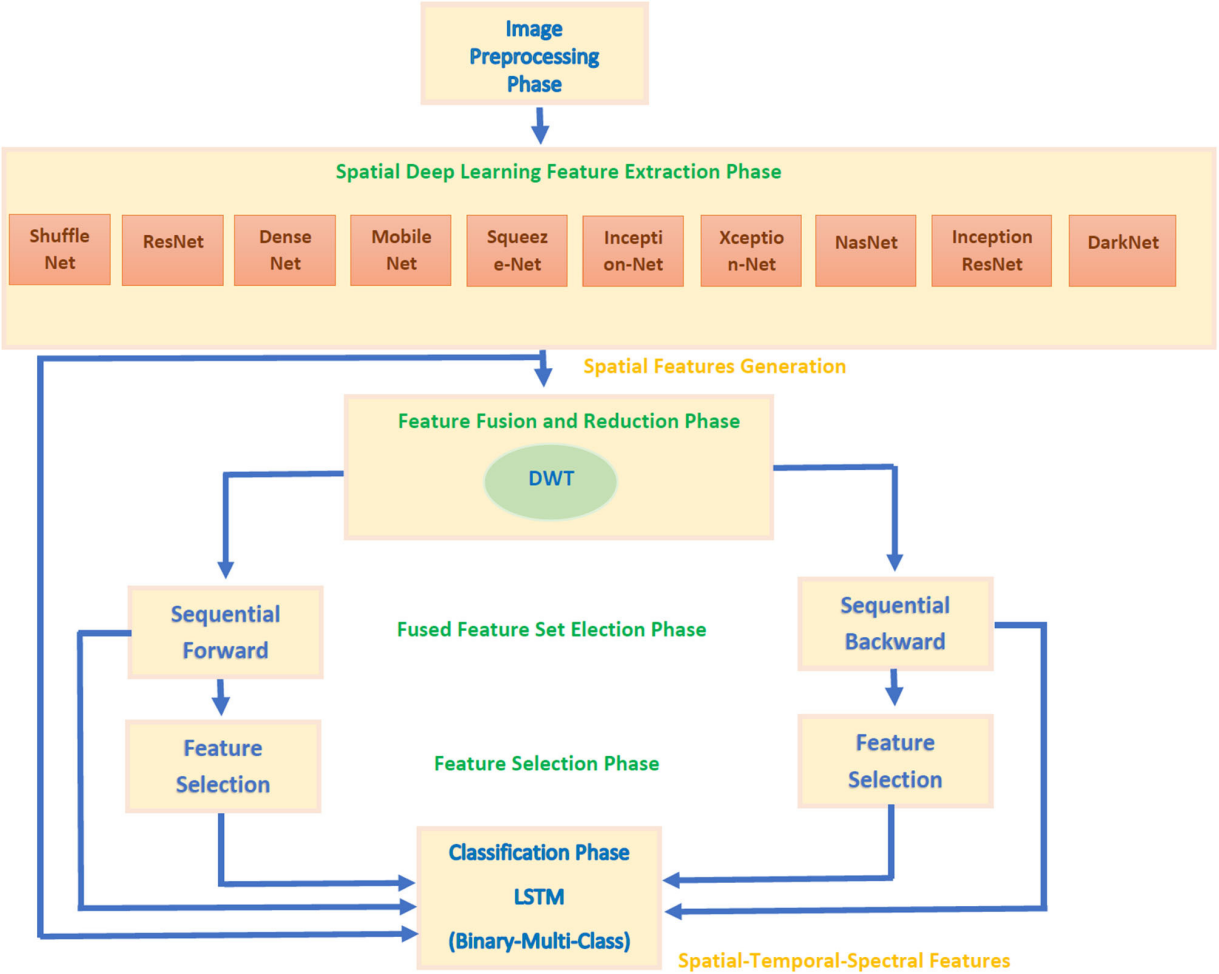
The block diagram of CoMB-Deep pipeline.

CoMB-Deep involves six phases: image preprocessing, deep spatial feature extraction, feature fusion and reduction, fused feature set election, feature selection, and classification phases. During the first phase, the microscopic images are augmented and resized. Afterward, spatial DL variables are obtained from 10 pre-trained CNNs in the deep spatial feature extraction phase. Next, these features are fused using the DWT method. DWT is also used in this phase to decrease the enormous size of fused features. Then, in the combined feature set election phase, the feature sets generated from the 10 CNNs are searched using two strategies to detect the most acceptable mixture of fused feature sets that influence the performance of CoMB-Deep. In the feature selection phase, two feature selection approaches are performed on the best-fused sets of features selected in the prior phase to lessen the size of fused features further. Finally, a bidirectional LSTM DL method is used to accomplish the classification procedure of both the binary and multi-class categories. Figure 2 illustrates the block diagram of the introduced CoMB-Deep.

#### Image Preprocessing Phase

During this phase, the microscopic images are resized to the input layer size of every CNN as shown in Table 1. Next, an augmentation step is made to enlarge the number of microscopic images available and avoid overfitting (Shorten and Khoshgoftaar, 2019). The techniques used for augmentation to create new microscopic scans from the training data in CoMB-Deep are translation (−30, 30), scaling (0.9, 1.1), flipping in x and y directions, shearing (0, 45) in x and y directions as observed by Ragab and Attallah (2020) and Attallah et al. (2020c).

**Table 1:** The depth, input, and output sizes of the 10 CNNs along with the description of the layers from which features where extracted.

**CNN structure**	**Depth**	**Size of input**	**Feature extraction layer name**	**Feature extraction layer description**	**Size of features**
Shuffle	50	224 × 224 × 3	“node 200”	The last average pooling layer just before the fully connected layer	544
ResNet-50	50	224 × 224 × 3	“avg_pool”	The last average pooling layer just before the fully connected layer	2,048
NasNetMobile	913	224 × 224 × 3	“global_average_pooling2d_1”	The last average pooling layer just before the fully connected layer	1,056
MobileNet	19	224 × 224 × 3	“avg_pool”	The last average pooling layer just before the fully connected layer	1,280
SqueezeNet	18	227x227x3	“relu_conv10”	Rectified Linear unit layer just after the 10th convolutional layer	392 (Binary)
					784(Multi-class)
DenseNet-201	201	224 × 224 × 3	“avg_pool”	The last average pooling layer just before the fully connected layer	1,920
Inception-V3	48	229 × 229 × 3	“avg_pool”	The last average pooling layer just before the fully connected layer	2,048
Xception	71	229 × 229 × 3	“avg_pool”	The last average pooling layer just before the fully connected layer	2,048
Inception-ResNet	164	229 × 229 × 3	“avg_pool”	The last average pooling layer just before the fully connected layer	1,536
DarkNet-53	53	256 × 256 × 3	“avg_1”	The last average pooling layer just before the fully connected layer	1,024

#### Deep Spatial Feature Extraction Phase

During this phase, spatial DL features are extracted from 10 CNNs architectures using transfer learning. These CNN architectures include Inception V3, Xception, ResNet-50, Inception-ResNet-V2, DenseNet-201, NasNetMobile, ShuffleNet, SqueezeNet, MobileNet, and DarkNet-53 networks. The transfer learning process corresponds to the ability of the network to identify similarities between distinct data, which helps develop the training procedure for a new similar classification task (Thrun and Pratt, 1998; Raghu et al., 2019). In other words, transfer learning enables pre-trained CNN to learn demonstration from a huge number of images, such as those available in ImageNet and afterward used this knowledge in a similar classification problem which has smaller datasets (Pan and Yang, 2009; Han et al., 2018). In this paper, 10 pretrained CNNs that were previously trained on the ImageNet dataset are employed. Transfer learning is also employed to extract deep spatial features from a specific layer of a CNN. In this phase, after adapting the FC layers and some of the parameters (discussed later in the parameter setting section) of the 10 pretrained CNNs to the number of labels in the dataset used in this paper rather than the 1,000 labels of ImageNet, the 10 networks are trained. Afterward, features are extracted from the layers as mentioned in Table 1. Table 1 illustrates the depth, input, and output sizes and mentions the layers where features are extracted.

#### Feature Fusion and Reduction Phase

Deep spatial features obtained in the earlier phase are fused using DWT. The reason for choosing DWT is that it is a well-known technique based on texture analysis and is commonly used in the medical area. DWT can reduce the vast dimension of the data and demonstrate temporal-frequency representations from any given data (Li and Meng, 2012; Ponnusamy and Sathiamoorthy, 2019). DWT utilizes a group of perpendicular basis functions called wavelets to analyze data (Antonini et al., 1992). In the case of 1-D data, like the deep spatial features mined from the 10 CNNs utilized in CoMB-Deep, the DWT process is performed by passing the data through low and high pass filters (Demirel et al., 2009). Afterward, a downsampling step is accomplished to reduce the data dimension (Hatamimajoumerd and Talebpour, 2019). Two sets of coefficients will be generated after this step: the approximation coefficients CA_1_ and detail coefficients CD_1_ (Attallah et al., 2019). This phase is executed to consider the privileges of DL and textural-based feature extraction approaches. The discrete Meyer wavelet (dmey) is employed as the mother wavelet. The CD_1_ variables are only selected because these coefficients comprise information from most of the data. Furthermore, the large size of the features extracted in the former phase is decreased.

#### Fused Feature Set Election Phase

Deep spatial variables mined in the deep spatial feature extraction phase are ranked in descending order according to the classification accuracy obtained using the LSTM network. This ranking is used to generate several feature sets that are fused using DWT. In this phase, two searching strategies, including the sequential backward and forward schemes (Attallah, 2020), are done to select the merged feature sets which have the highest impact on the accuracy attained by CoMB-Deep. In the backward scheme, the feature set selection begins with fusing all features using DWT and eliminating the lowest ranking feature (lowest accuracy). Next, each set of features having a better ranking is deleted; if the excluded set of features improved the accuracy of CoMB-Deep, it is kept deleted; else, it is not deleted. Alternatively, in the forward scheme (Fauvel et al., 2015; Pohjalainen et al., 2015), the selection begins with the first feature set having the highest rank. Afterward, every successive set of features is included one by one; if the set of features included increased the accuracy of CoMB-Deep, it is retained; else, it is ignored. This procedure continues until all feature sets are investigated (Ververidis and Kotropoulos, 2005).

#### Feature Selection Phase

To further reduce the vast dimension of the fused feature sets chosen in the previous phase, feature selection is essential (Attallah et al., 2017b) because the massive size of the features raises the complexity of the classification phase and might decrease its performance (Li and Liu, 2017; Hatamimajoumerd et al., 2020). Feature selection is frequently utilized in medical frameworks to reduce the dimension of the feature set and delete unnecessary and irrelevant variables (Chandrashekar and Sahin, 2014; Attallah et al., 2017a, 2020a; Cai et al., 2018). In this phase, two feature selection approaches, including Relief-F and Information gain (IG) feature selection methods are used to select a reduced set of features.

*Information gain* is a standard filter feature selection method. The fundamental concept of filter methods is to utilize the overall characteristics of the data to choose the most significant attributes based on these characteristics before the construction process of the classifier. The main benefit of the filter feature selection approach is its fast and straightforward feature (Sánchez-Marono et al., 2007). IG selects relevant features according to the fundamental idea of the entropy by calculating the differences among the entropy of the whole training samples and the weighted total sum of the values of the subset of their partition (classes) available for a given attribute (Roobaert et al., 2006). IG method is considered to be one of the fastest and straightforward filter methods. Therefore, it is utilized in this paper.

*Relief-F feature selection* is a well-recognized filter feature selection approach commonly used in medical classification problems due to computation efficiency and straightforwardness. The Relief-F method was proposed by Kononenko (1994) to be used for multi-class, noisy, and incomplete problems of the datasets. Its basic idea is to calculate the significance of features based on their capabilities to differentiate among the same class instances that are close and distinct to others in a local neighborhood. This capability is measured by estimating the weight or score of each feature (Urbanowicz et al., 2018). Those features that have a higher ability to distinguish between different class instances and increase the distance between them are given higher scores than others with lower capacities.

#### Classification Phase

**Figure 3:**
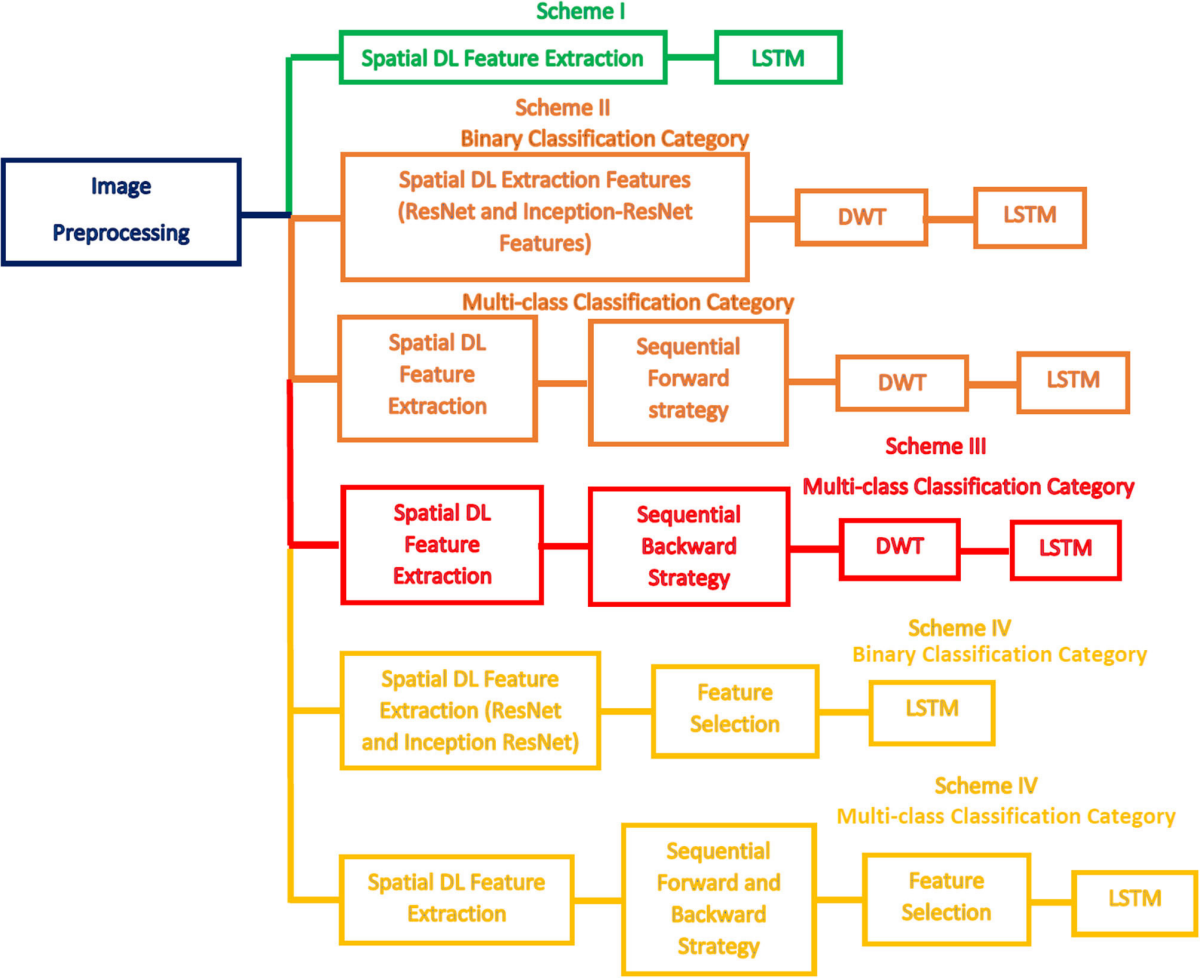
The four schemes of CoMB-Deep.

The Bi-LSTM DL-based technique is used in the two classification categories of CoMB-Deep (binary and multi-class). This process is accomplished using four schemes. In the first scheme, spatial DL features are taken out from the 10 CNNs to train several Bi-LSTM classifiers. Afterward, these spatial DL features are ranked according to the accuracy achieved by the Bi-LSTM. This ranking is used in the following two schemes. The order of the previous scheme is employed to explore the most acceptable combination of fused features during the second scheme, enhancing the accuracy of the LSTM classifier using a sequential forward strategy. Note that fusion is done using the DWT method. The third scheme is like the second scheme, but it uses a sequential backward strategy instead of a sequential forward strategy. In the last scheme, two feature selection approaches are used to select a reduced number of features from the chosen fused feature sets in the second and third schemes. Figure 3 defines the four schemes of the proposed CoMB-Deep.

All CoMB-Deep schemes were done with Matlab 2020a. The Bi-LSTM classifier was constructed using the Weka data mining software (Hall et al., 2009). The processor used is Intel(R) Core (TM) i7-10750H with NVIDIA GeForce GTX 1660 video controller of 6 GB capacity, with the processor frequency of 2.6 GHz 64-bit operating system.

## Experimental Settings

### Assessment Measures

To assess the performance of the presented CoMB-Deep pipeline, several assessment measures are utilized. These measures include the accuracy, Matthews correlation coefficient (MCC), sensitivity, precision, F1 score, and specificity. They are computed using the following rules (Attallah et al., 2020b) (Equations 1–6).

(1) $$\begin{array}{l} Accuracy=\frac{TP+TN}{TN+FP+FN+TP} \end{array}$$

(2) $$\begin{array}{l} Sensitivity=\frac{TP}{TP+FN} \end{array}$$

(3) $$\begin{array}{l} Specificity=\frac{TN}{TN+FP} \end{array}$$

(4) $$\begin{array}{l} Precision=\frac{TP}{TP+FP} \end{array}$$

(5) $$\begin{array}{l} MCC=\frac{TP\times TN-FP\times FN}{\sqrt{\left(TP+FP\right)\left(TP+FN\right)\left(TN+FP\right)\left(TN+FN\right)}} \end{array}$$

(6) $$\begin{array}{l} F1-Score=\frac{2\times TP}{\left(2\times TP\right)+FP+FN} \end{array}$$

Where, the number of MB images that are correctly identified to belong to the MB class which they refer to is called true positive (TP) and true negative (TN) is the total number of MB images that does not refer to the identified MB class, and does not actually refer to. For each subtype of MB tumor, false positive (FP) is the sum of all classified images as this MB subtype, but they do not truly belong to. For each subtype of MB tumor, false negative (FN) is the total sum of images not classified as this MB subtype.

### Parameters Set Up

Several parameters are modified for the 10 CNNs. The mini-batch dimension and validation frequency are set to 4 and 26 for multi-class and 17 for binary class. The epochs and the initial learning rate are found to be 20 and 3 × 10^−4^, respectively, while other parameters of the 10 CNNs are set with their default values. To assess the performance of the classification process and avoid overfitting, a 10-fold cross-validation is applied. It is used in Bi-LSTM classification, and the announced accuracy is found to be average across the 10-cross validation folds. For the Bi-LSTM network, the number of epochs is set to 30, the batch size is set to 100, and the validation frequency is set to 10.

## Results

This section will illustrate the four scheme results of the CoMB-Deep pipeline for the binary and multi-class classification categories. Scheme I represents the deep spatial features obtained through the 10 CNNs distinctly as inputs to LSTM networks. The classification accuracies attained using the 10 DL features are utilized to rank them in the descending order. This sorting is then employed in the following two schemes. Scheme II explores the 10 spatial feature sets using a forward strategy to determine the best combined spatial feature sets that improve the performance of CoMB-Deep. Similarly, Scheme III searches for the most acceptable mixture of combined feature sets but using a backward strategy. The combination of the feature sets is made utilizing DWT to reduce the dimension of the fused feature sets. In Scheme IV, two feature selection methods are employed to further decrease the size of the merged feature sets and to select a reduced number of features.

### Results of Scheme I

The results of Scheme I are shown in Table 2. For the binary classification category, the highest accuracy of 100% is attained using Inception-ResNet-V2 and ResNet-50. This is followed by DarkNet 53, Inception V3, MobileNet, and Squeeze CNNs to obtain an accuracy of 99%. Then, DenseNet-201 and ShuffleNet CNNs accomplish an accuracy of 98%, which is >96 and 92% accuracy obtained using Xception and NasNetMobile CNNs. On the other hand, for the multi-class classification category, the greatest accuracy of 96.1% is achieved using DenseNet-201 and Inception V3 spatial features. Next, ShuffleNet, SqueezeNet, and ResNet-50 features reach an accuracy of 95.45, 92.86, and 92.2%, respectively. Afterward, both Inception-ResNet-V2 and MobileNet features attain an accuracy of 90.91%. Subsequently, DarkNet-53, Xception, and NasNetMobile CNNs obtain accuracies of 88.31, 87.66, and 75.97%.

**Table 2:** The classification accuracies (%) achieved using LSTM network trained with individual DL features for binary and multi-class classification categories.

**CNN**	**Binary classification**	**Multi-class classification**
DarkNet53	99	88.31
DenseNet-201	98	96.1
Inception	99	96.1
InceptionResNet	100	90.91
MobileNet	99	90.91
NasNetMobile	92	75.97
ResNet-50	100	92.2
ShuffleNet	98	95.45
Xception	96	87.66
Squeeze	99	92.86

### Results of Scheme II

This section discusses the results of the fusion of multiple DL features using the forward strategy and DWT. Note that in the binary classification category, it can be noticed that both the Inception-ResNet-V2 and ResNet-50 attain a 100% accuracy in Scheme I; thus, there is no need for feature fusion in the binary classification category done in Schemes II and III. The DWT in this category is used to reduce the dimension of features extracted from both Inception-ResNet-V2 and ResNet-50. Table 3 shows the accuracy and size of features after and before the DWT for both Inception-ResNet-V2 and ResNet-50 CNNs. It is clear from Table 3 that the DWT has reduced the number of features from 2,048 to 1,074 for the ResNet-50 CNN and from 1,536 to 818 features for the Inception-ResNet-V2 achieving the same accuracy of 100%.

**Table 3:** Binary classification accuracy (%) and number of features before and after DWT for ResNet-50 and Inception-ResNet CNNs.

**Type of features**	**No of features**	**Accuracy**
**ResNet-50**		
Spatial DL	2,048	100
DWT	1,074	100
**Inception-ResNet**		
Spatial DL	1,536	100
DWT	818	100

**Figure 4:**
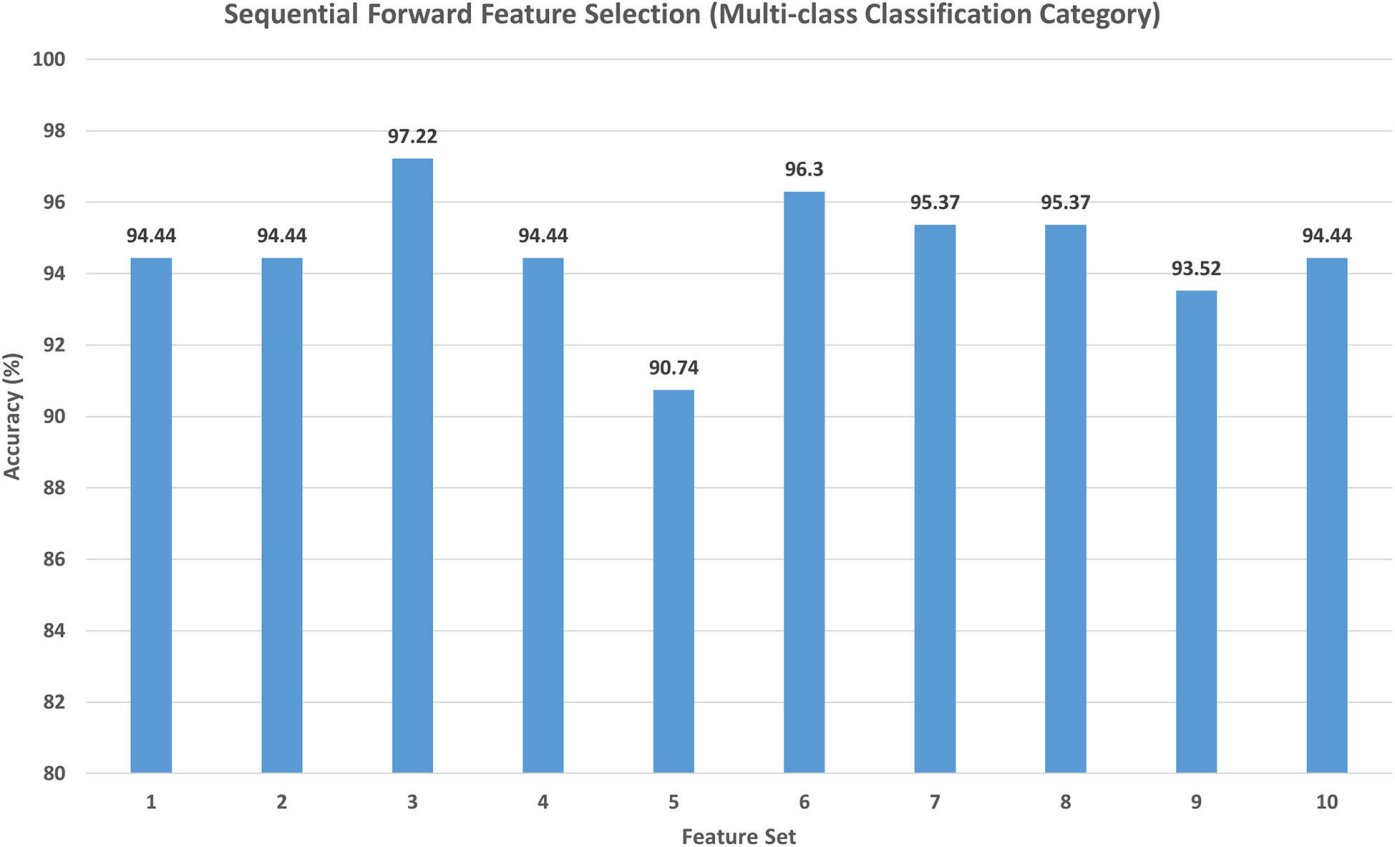
The classification accuracy (%) attained for the different feature sets generated using forward strategy. Feature set 1: DenseNet-201 (1,010 Features); Feature set 2: DenseNet-201 + Inception V3(2,034 Features); Feature set 3: DenseNet-201 + Shuffle (1,282 Features); Feature set 4: DenseNet-201 + Shuffle + Squeeze (1,674 Features); Feature set 5: DenseNet-201 + Shuffle + ResNet-50 (2,306 Features); Feature set 6: DenseNet-201 + Shuffle + MobileNet (1,922 Features); Feature set 7: DenseNet-201 + Shuffle + Inception-ResNet-V2 (2,050 Features); Feature set 8: DenseNet-201 + Shuffle + DarkNet-53 (1,794 Features); Feature set 9: DenseNet-201 + Shuffle + Xception (2,306 Features); Feature set 10: DenseNet-201 + Shuffle + NasNetMobile (1,810 Features).

For the multi-class classification category, the main target of Scheme II is to explore the different combinations of deep spatial features using the forward strategy. The search starts with the feature set 1 corresponding to only one feature type. It starts to add feature sets iteratively; if the feature set included increased the accuracy, then it is kept, else it is ignored. Note that the feature selection processing is performed on the training set separate from the testing set. The feature set selection results on the training set of the multi-class classification category of Scheme II are shown in Figure 4. This figure shows that feature set 3, which contains the fused features of DenseNet-201+ShuffleNet after DWT having a dimension of 1,282 features, has attained the highest accuracy of 97.22%. For this reason, it is selected in Scheme II. The results of feature set 3 are then evaluated on a separate testing set, and the results are shown in Table 4. Table 4 indicates that feature set 3 selected using the forward strategy has an accuracy of 95.65%, a sensitivity of 0.957, a specificity of 0.992, a precision of 0.958, an F1-score of 0.946, and an MCC of 0.99.

**Table 4:** Testing performance metrics of the feature sets selected using forward and backward strategies.

**Selected feature set**	**Accuracy (%)**	**Sensitivity**	**Specificity**	**Precision**	**F1-score**	**MCC**
**Forward search strategy**						
Feature Set 3	95.65	0.957	0.992	0.966	0.958	0.946
**Backward search strategy**						
Feature Set 5	95.65	0.957	0.975	0.961	0.955	0.940

### Results of Scheme III

**Figure 5:**
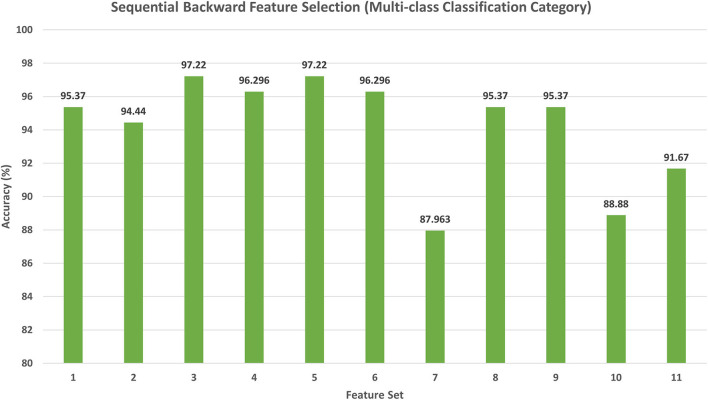
The classification accuracy (%) attained for the different feature sets generated using backward strategy. Feature set 1: DenseNet-201 + Inception V3 + Shuffle + Squeeze + ResNet-50 + MobileNet + Inception-ResNet-V2 + DarkNet-53 + Xception + NasNetMobile (7,194 Features); Feature set 2: DenseNet-201 + Inception V3 +Shuffle + Squeeze + ResNet-50 +MobileNet + Inception-ResNet-V2 + DarkNet-53 +Xception (6,666 Features); Feature set 3: DenseNet-201 + Inception V3 + Shuffle + Squeeze + ResNet-50 + MobileNet + Inception-ResNet-V2 + DarkNet-53 + NasNetMobile (6,170 Features); Feature set 4: DenseNet-201 + Inception V3 + Shuffle + Squeeze + ResNet-50 + MobileNet + Inception-ResNet-V2 + NasNetMobile (5,658 Features); Feature set 5: DenseNet-201 + Inception V3 +Shuffle + Squeeze + ResNet-50 + MobileNet+DarkNet-53 + NasNetMobile (5,402 Features); Feature set 6: DenseNet-201 + Inception V3 + Shuffle + Squeeze + ResNet-50 + DarkNet-53 + NasNetMobile (4,762 Features); Feature set 7: DenseNet-201 + Inception V3 + Shuffle + Squeeze + MobileNet + DarkNet-53 + NasNetMobile (4,378 Features); Feature set 8: DenseNet-201 + Inception V3 +Shuffle + ResNet-50 + MobileNet+DarkNet-53 + NasNetMobile (5,010 Features); Feature set 9: DenseNet-201 +Inception V3 + Squeeze + ResNet-50 + MobileNet + DarkNet-53 + NasNetMobile (5,030 Features); Feature set 10: DenseNet-201 + Shuffle + Squeeze + ResNet-50 + MobileNet + DarkNet-53 + NasNetMobile (4,378 Features); Feature set 11: Inception V3 + Shuffle + Squeeze + ResNet-50 + MobileNet + DarkNet-53 + NasNetMobile (4,442 Features).

The results of the backward search strategy for the multi-class classification category are illustrated in this section. Feature set 1 corresponds to the fusion of all the 10 deep features; each feature set is eliminated iteratively; if the accuracy is improved, then it is removed; else, it is kept. The results of the backward strategy executed on the training set are shown in Figure 5. It can be noticed from Figure 5 that feature sets, 3 and 5 achieve the highest accuracy of 97.22%. Feature set 3 has a dimension of 6,170 features representing the fusion of the features from all CNNs except those of Xception, whereas feature set 5 contains 5,402 features of the combined DenseNet-201 + Inception V3 + ResNet-50 + Darknet-53 + MobileNet + ShuffleNet + SqueezeNet + NasNetMobile CNNs. This dimension shows that feature set 5 has a lower size of features than the feature set 3; therefore, it is selected. The testing performance of feature 5 selected during the backward strategy is shown in Table 4. Table 4 illustrates that feature set 5 chosen using backward strategy has an accuracy of 95.65%, a sensitivity of 0.957, a specificity of 0.975, a precision of 0.961, an F1-score of 0.955, and an MCC of 0.94.

### Results of Scheme IV

This section illustrates the results of Relief-F and IG feature selection methods for both classification categories of CoMB-Deep. Table 5 shows the number of features, classification accuracy, and other performance metrics after feature selection for the binary classification category. This table verifies that 100% accuracy is achieved using only 578 features selected using Relief-F and IG methods instead of 1,074 utilized in Scheme II (using DWT) for ResNet-50 CNN. On the other hand, the same accuracy is reached using 200 and 550 features obtained with Relief-F and IG approaches which are lower than the 818 features employed in Scheme II (using DWT) Inception-ResNet-V2 CNN. It is clear from the table that the sensitivities, specificities, precisions, F1-scores, and MCCs are equal to 1.

**Table 5:** Binary classification accuracy (%), number of features, and other performance metrics before and after feature selection for ResNet-50 and Inception-ResNet CNNs.

**Method**	**No of features**	**Accuracy (%)**	**Sensitivity**	**Specificity**	**Precision**	**F1-score**	**MCC**
**ResNet-50**							
DWT	1,074	100	1	1	1	1	1
Relief-F	578	100	1	1	1	1	1
IG	578	100	1	1	1	1	1
**Inception-ResNet**							
DWT	818	100	1	1	1	1	1
Relief-F	200	100	1	1	1	1	1
IG	550	100	1	1	1	1	1

For the multi-class classification category, it can be noticed from Table 6 that for the forward search strategy, both Relief-F and IG methods have reduced the number of features from 1,282 features of feature set 3 (Scheme II) to 448 and 738 features, respectively, while attaining the same accuracy of 98.05% which is higher than that obtained in Scheme II. Similarly, in the backward search strategy, the features selected using Relief-F and IG methods have attained an accuracy of 99.35%, which is better than that obtained in Scheme III, where the number of features has been decreased to 739 (for Relief-F), and 2,313 (for IG) which are lower than the 5,403 features of feature set 5 chosen in Scheme III. Table 6 also indicates that both feature selection methods for the forward strategy achieve a sensitivity of 0.981, precisions of 0.981, and F1 scores of 0.981 and 0.972, respectively. The specificity and MCC attained using Relief-F, and IG methods are 0.993, 0.99, 0.972, and 1. For the backward approach, both feature selection methods achieved sensitivities of 0.994, specificities of 0.996, precisions of 0.984, F1 scores of 0.993, and MCCs of 0.991.

**Table 6:** Binary classification accuracy (%), number of features, and other performance metrics before and after feature selection for the feature set 5 selected using forward and backward strategies.

**Method**	**No of features**	**Accuracy (%)**	**Sensitivity**	**Specificity**	**Precision**	**F1-score**	**MCC**
**Forward search**							
Relief-F	448	98.05	0.981	0.993	0.981	0.981	0.972
IG	738	98.05	0.981	0.99	0.981	0.972	1
**Backward search**							
Relief-F	739	99.35	0.994	0.996	0.994	0.993	0.991
IG	2,313	99.35	0.994	0.996	0.994	0.993	0.991

## Discussion

This study presented a computer-assisted pipeline called CoMB-Deep for the automatic classification of childhood MB and its classes from histopathological images. CoMB-Deep has two classification categories: binary and multi-class classification categories. CoMB-Deep discriminates between normal and MB images in the binary classification category, whereas the multi-class classification category classifies the four classes of childhood MB. The presented pipeline involved a mixture of deep learning techniques fused by the DWT method. CoMB-Deep searched for the most acceptable combination of combined deep learning feature sets using forward and backward strategies. It consists of six phases which include image preprocessing, deep spatial feature extraction, feature fusion and reduction, fused feature set election, feature selection, and classification phases. In the first phase, images were resized and augmented, and then spatial deep features were extracted from 10 CNNs. Afterward, a fusion process was performed using the DWT method, which lowered the dimension of the fused features. Next, sequential forward and backward search strategies were utilized to explore the best-reduced feature sets, which improved the accuracy of CoMB-Deep. Two feature selection approaches were then employed further to reduce the selected feature sets of the previous phase. Lastly, in the classification phase, a bidirectional LSTM classifier was utilized to accomplish classification.

CoMB-Deep consists of a cascaded composite of CNNs and Bi-LSTM. For the feature extraction, these composite of CNNs extracted the spatial sequence of features (N) from the images. Afterward, these features were fused using the DWT method, which presents the time-frequency representation of the features. Next, the Bi-LSTM (Hochreiter and Schmidhuber, 1997) was employed to learn the temporal information from the set of N features vectors previously extracted and fused using the CNNs and DWT. The Bi-LSTM is an extension of a bidirectional RNN (Schuster and Paliwal, 1997) that is capable of modeling the temporal dependent states, and this is done through a direct cyclic connection among its units that can save its intrinsic hidden status and enable the modeling of the dynamic temporal attitude. It consists of three gates called input, output, and forget gates, which help realize the prolonged temporal dependencies.

**Figure 6:**
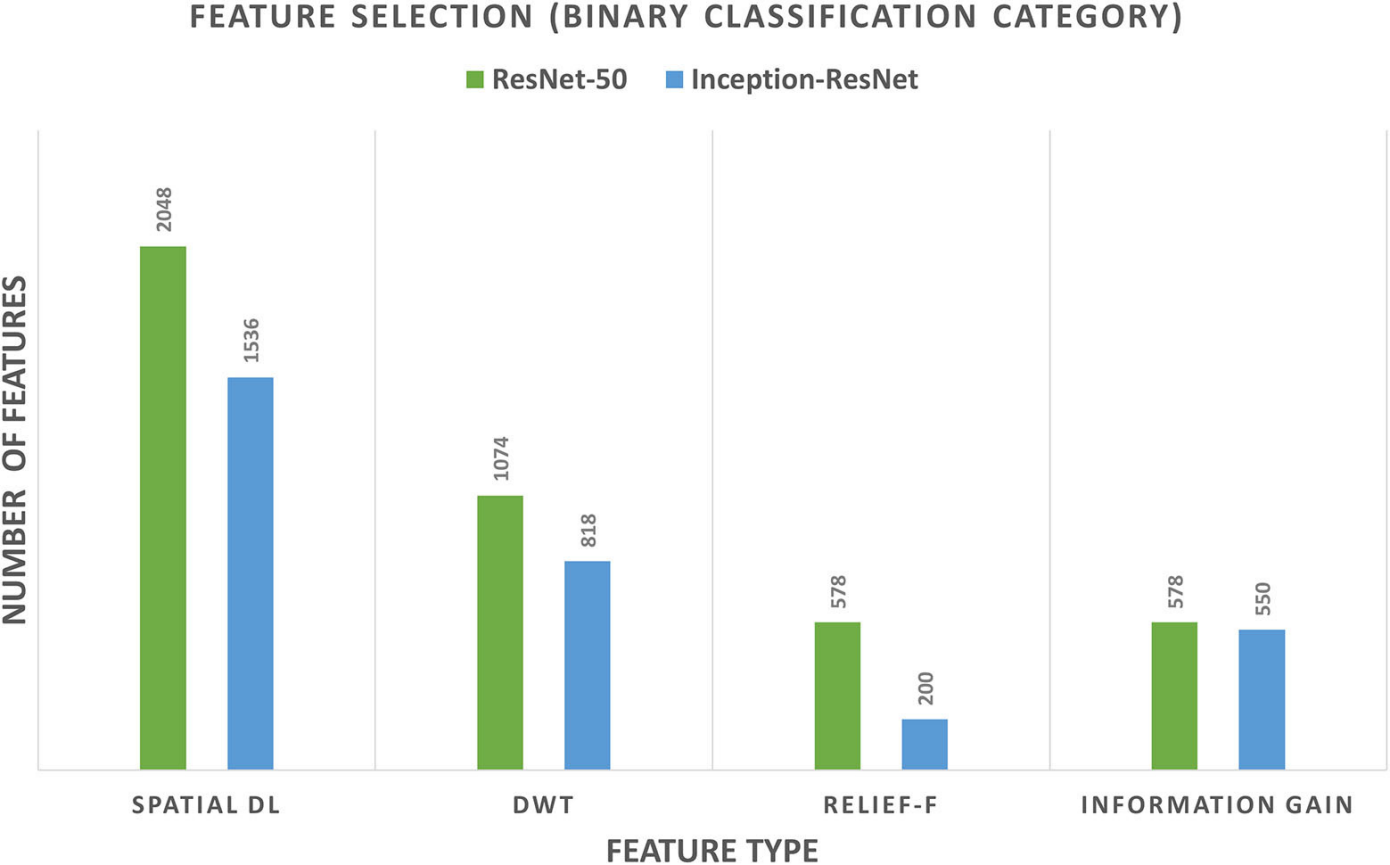
The number of features of Scheme I (spatial DL feature extraction), feature reduction (DWT), and the Relief-F and information gain feature selection methods for both Inception-ResNet-V2 and ResNet-50 CNNs for the binary classification category.

For the binary classification category of CoMB-Deep, it was noticed from the results of Scheme I that both Inception-ResNet-V2 and ResNet-50 had attained a 100% accuracy. Therefore, feature fusion using forward and backward strategies were not performed. The feature reduction process using DWT followed by the Relief-F and IG feature selection methods was only accomplished. Figure 6 shows the number of features of Scheme I (spatial DL feature extraction), feature reduction (DWT), the Relief-F, and IG feature selection methods for both Inception-ResNet-V2 and ResNet-50 CNNs. The figure shows that the number of features of Scheme I is 2,048 and 1,536 for ResNet-50 and Inception-ResNet-V2, respectively. The DWT technique has reduced the number of features to 1,074 and 818 for ResNet-50, and Inception-ResNet-V2 DL features, respectively. These features were further reduced to 578 and 200 using the Relief-F approach, and 578 and 550 using the IG method for ResNet-50 and Inception-ResNet-V2 DL features. Note that the accuracy attained after all these processes is 100%. This means that the CoMB-Deep has successfully decreased the number of features used to construct the Bi-LSTM classifier while attaining the same accuracy of 100%.

**Figure 7:**
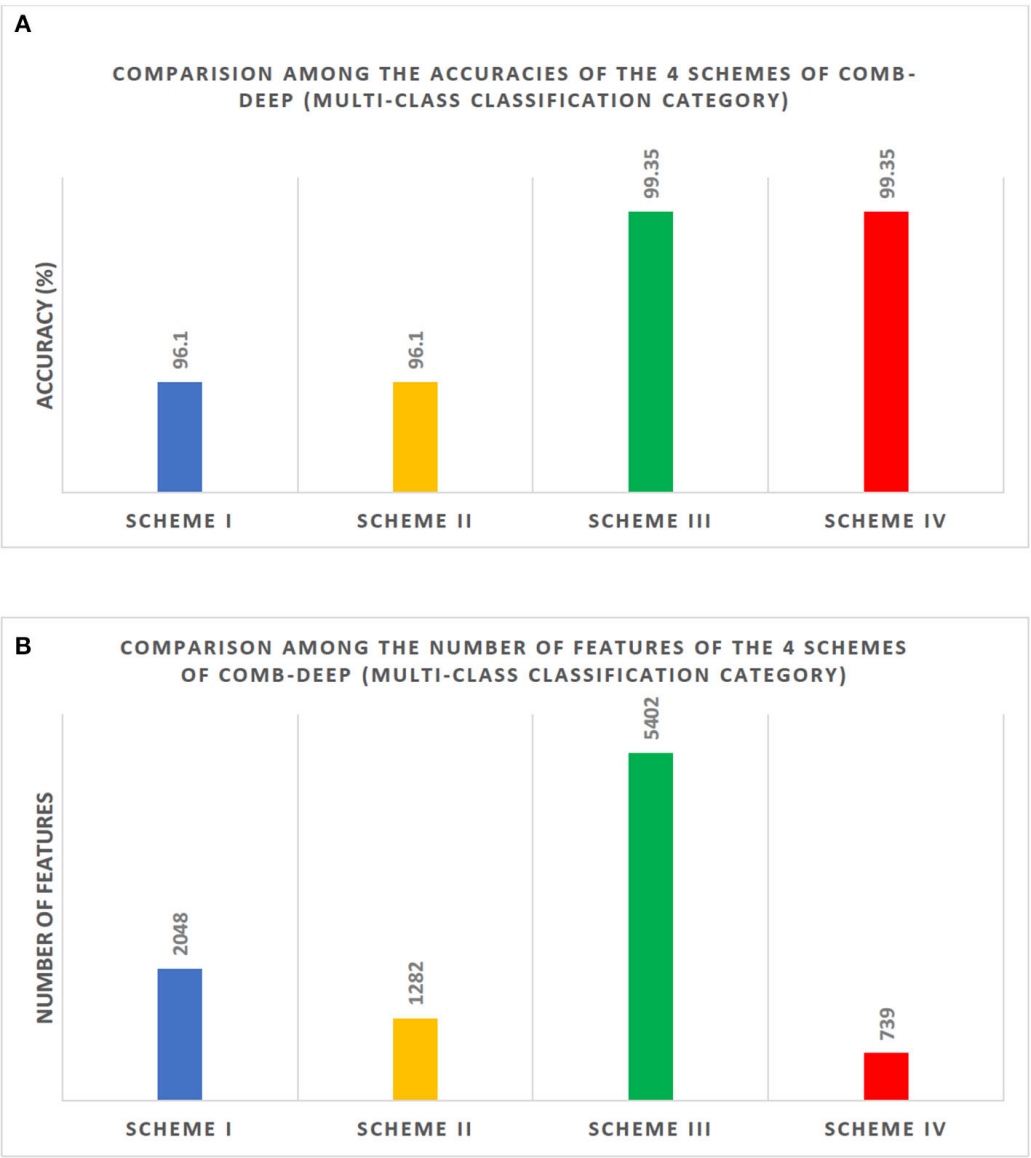
Comparison between the four schemes of the multi-class classification category of CoMB-Deep. **(A)** The highest classification accuracy (%) attained using the four schemes. **(B)** The number of features utilized in the four schemes. Scheme I: ResNet-50 or DenseNet-201; Scheme II: DenseNet-201 + Shuffle; Scheme III: DenseNet-201 + Inception V3 + Shuffle + Squeeze + ResNet-50 + MobileNet + DarkNet-53 + NasNetMobile; Scheme IV: DenseNet-201 + Inception V3 + Shuffle + Squeeze + ResNet-50 + MobileNet + DarkNet-53 + NasNetMobile.

On the other hand, for the multi-class classification, Figure 7A shows a comparison between the highest classification accuracies attained using the four schemes of CoMB-Deep using a 10-fold cross-validation. The figure indicates that the highest accuracy of 96.1% is obtained using ResNet-50 and DenseNet-201 features in Scheme I. For Scheme II, the same accuracy of 96.1% is obtained using feature set 3 (Dense-201 + Shuffle Features). The figure also verifies that Scheme III (Feature set 5 for backward search strategy without feature selection), including DenseNet-201 + Inception V3 + Shuffle + Squeeze + ResNet-50 + MobileNet + DarkNet-53 + NasNetMobile, and Scheme IV (Feature set 5 for backward search strategy with feature selection) have higher accuracies than the previous schemes. The highest accuracy attained using both Schemes III and IV is 99.35%. The peak accuracy in Scheme II (forward search strategy) was achieved using the feature set 3 corresponding to feature size of 1,282 features as shown in Figure 7B. The maximum accuracy in Scheme III was reached using feature set 5 (backward strategy), representing 5,402 features as illustrated in Figure 7B. In contrast, Scheme IV has lowered the feature dimension to 739 features using the Relief-F feature selection technique. Figure 7B compares the number of features obtained using the four schemes of CoMB-Deep.

**Figure 8:**
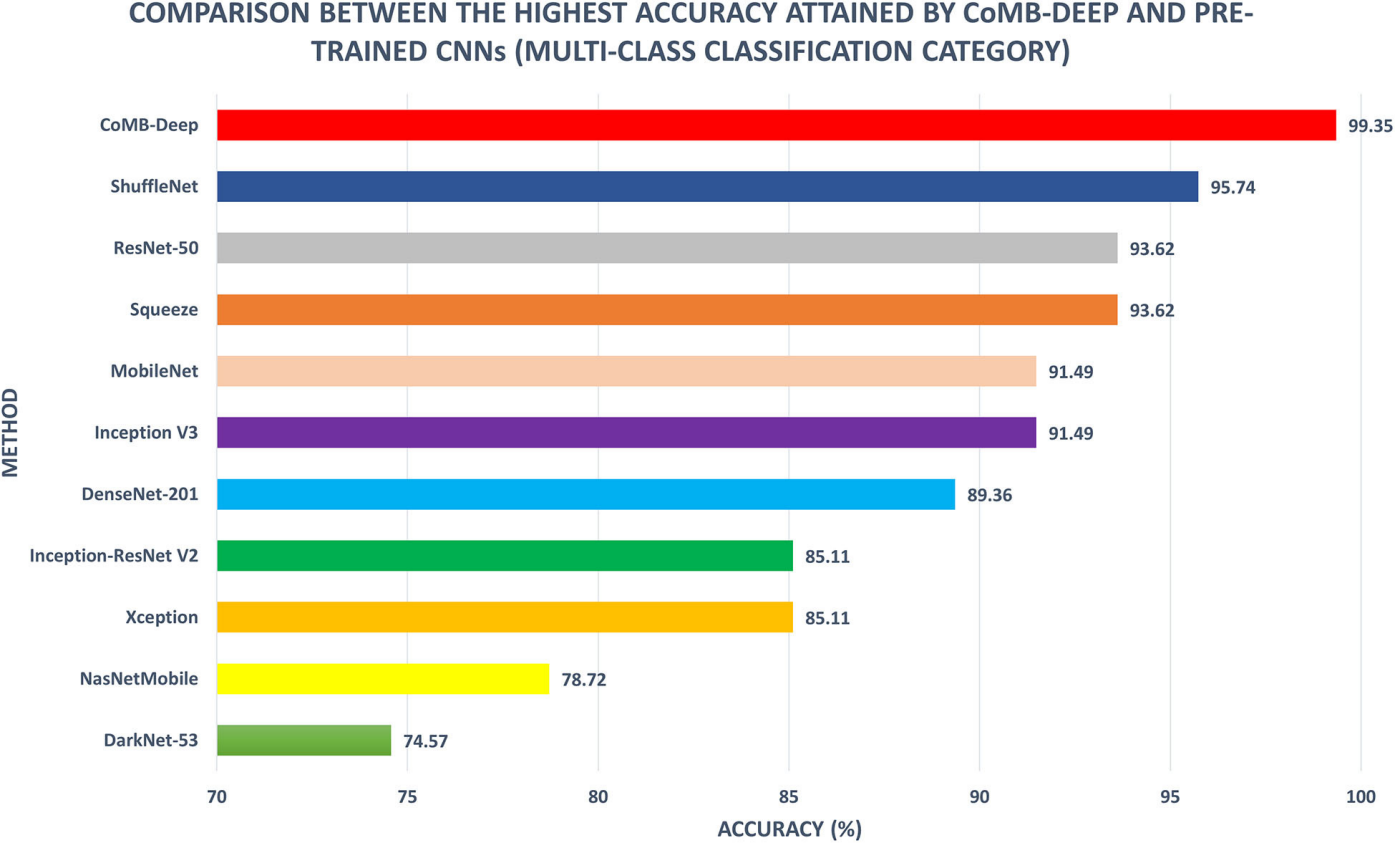
Multi-class classification accuracy (%) of CoMB-Deep compared to end-to-end deep learning classification of 10 CNNs.

Figure 8 shows a comparison between the classification accuracy of CoMB-Deep compared to the end-to-end DL classification of the 10 CNNs for the multi-class classification category. It is evident from the figure that CoMB-Deep has outperformed the 10 CNNs constructed based on an end-to-end classification procedure. This is because CoMB-Deep has attained an accuracy of 99.35% using 1,541 features selected using the IG method (Scheme IV) applied on the feature set 5 chosen using the forward search strategy (Scheme II). The outperformance of CoMB-Deep proves that it is better than using individual CNNs constructed by an end-to-end approach. This verifies that CoMB-Deep can help the pathologist classify the four childhood MB classes, which can help them select the appropriate treatment and follow-up plan.

The performance of CoMB-Deep is compared with related studies based on the same dataset to prove its competence. Table 7 displays this comparison. Das et al. (2018b) extracted textural features, comprising GLCM, HOG, Tamura, LBP, and GLCM. They fused all these features and used PCA to reduce them and SVM classifier to classify pediatric MB classes. The highest accuracy attained was 84.9%. Similarly, the same authors utilized the same textural features and fused them all using MANOVA. The authors employed SVM for classification, attaining an accuracy of 65.2%. It can be noticed from these results that fusing all these features does not always guarantee the best performance. Therefore, the authors (Das et al., 2020b) searched for the best combination of these feature extraction methods and found that fusing only four features sets (GLCM + Tamura + LB + GRLN) has the highest impact on the classification performance, obtaining an accuracy of 91.3% using SVM classifier and 96.7% using PCA with SVM classifier. This means that investigating a different combination of feature sets and selecting the most influential fused feature set can improve the accuracy of the classifier. In the same manner, CoMB-Deep pipeline explored different combinations of features extracted from several CNNs and fused using DWT to select the most fused feature sets that impact the performance of the classifier As mentioned before DL techniques are more favorable than the traditional feature extraction methods (Das et al., 2018b, 2020a,b). CoMB-Deep merges DL and textural analysis benefits, as it first extracts spatial features from 10 CNNs. It then searches for the most significant feature sets fused using DWT producing spatial-temporal-frequency features. DWT is a textural analysis technique that illustrates the temporal-frequency information of the data while reducing the dimension of the features. Finally, the Bi-LSTM DL technique is used for classification. The accuracy attained by CoMB-Deep is 99.35% which proves that merging DL techniques is better than conventional feature extraction techniques as it is higher than those achieved by Das et al. (2018b, 2020a,b). Also, it verifies that combining DL and textural analysis feature extraction methods can enhance the classification accuracy. Furthermore, utilizing spatial-temporal-frequency features is better than using each one of them independently.

**Table 7:** A comparison between CoMB-Deep and related studies using the same dataset.

**Classification category**					
	**Method**	**Sensitivity (%)**	**Precision (%)**	**Specificity (%)**	**Accuracy (%)**
Das et al. (2019)	HOG, GLCM, Tamura, and LBP features + GRLN + SVM	100	100	100	100
Das et al. (2020c)	AlexNet + SVM VGG-16 + SVM	–	–	–	99.44 99.62
Das et al. (2018b)	(Shape + Color) features + PCA + SVM	100	100	100	100
Das et al. (2020c)	AlexNet sVGG-16	–	–	–	98.5 98.12
Das et al. (2020a)	HOG, GLCM, Tamura, and LBP features + GRLN + MANOVA + SVM	100	100	100	100
Proposed CoMB-Deep	Inception-ResNet + DWT + Information gain + LSTM	100	100	100	100
**Classification (multi-class category)**					
		**Sensitivity (%)**	**Precision (%)**	**Specificity (%)**	**Accuracy (%)**
Das et al. (2018b)	(Shape + Color) features + PCA + SVM	–	–	–	84.9
Das et al. (2020a)	HOG, GLCM, Tamura, and LBP features + GRLN + MANOVA + SVM	72	66.6	–	65.21
Das et al. (2020c)	AlexNet VGG-16 s	–	–	–	79.33 65.4
Das et al. (2020b)	GLCM + Tamura + LBP + GRLN + SVM	91.3	91.3	97	91.3
Das et al. (2020b)	GLCM + Tamura + LBP + GRLN + PCA + SVM	–	–	–	96.7
Das et al. (2020c)	AlexNet + SVM VGG-16 + SVM	–	–	–	93.21 93.38
Proposed CoMB-Deep	Deep features of (DenseNet-201 + ShuffleNet) + Relief-F + Bi-LSTM	98.1	98.1	99.3	98.05
	Deep features of (DenseNet-201 + Inception + Resnet-50 + Darknet-53 + MobileNet + ShuffleNet + SqueezeNet + NasNetMobile) + Relief-F + Bi-LSTM	99.8	99.4	99.4	99.35

The results indicated in Table 7 verify that CoMB-Deep is a competitive pipeline for both classification categories. This is obvious as CoMB-Deep accomplished an accuracy of 100% (for binary classification category) which is the same as observed by Das et al. (2018b, 2019, 2020a), but greater than that achieved by Das et al. (2020c). The competitiveness of CoMB-Deep is found in its ability to distinguish the subclasses of MB. This is because it reached an accuracy of 98.05%, a sensitivity of 0.981, a specificity of 0.993, and a precision of 0.981 using the forward strategy in Scheme IV. It also reached 99.35% accuracy, a sensitivity of 0.998, specificity, and precision of 0.994 using the backward strategy in Scheme IV. These performance metrics achieved in Scheme IV are greater than all related works. This outperformance confirms that CoMB-Deep is reliable; thus, it may be utilized to support clinicians and pathologists in performing diagnosis with high accuracy, decreasing the chances of misdiagnosis during manual diagnosis, and speeding up the classification process, and finally, lower the cost of diagnosis.

## Conclusion

This study introduced a computer-assisted pipeline called CoMB-Deep, a composite of deep learning techniques for the automatic classification of MB and its classes. It has six phases: image preprocessing, spatial DL feature extraction, feature fusion and reduction, fused feature set election, feature selection, and classification phases. CoMB-Deep examined if deep feature fusion can enhance the accuracy of Bi-LSTM classifier compared to deep individual features. The fusion phase was accomplished using DWT, which lowers the dimension of fusion features, whereas deep feature sets are explored using forward and backward search methods. The results showed that deep feature fusion had improved the performance of the Bi-LSTM classifier. To further reduce the dimension of selected features chosen using forward and backward approaches, Relief-F and information gain feature selection methods were utilized. The results verified that both methods had successfully decreased the dimension of features while attaining the same peak accuracy of 100% and 99.35% for binary and multi-class classification categories, respectively. The performance of CoMB-Deep was compared with related works, which proved its competence; thus, it is reliable and can be used to help pathologists in accurately classifying childhood MB and its classes. Also, it may reduce the complications that pathologists face during manual diagnosis. It can accelerate the classification procedure while achieving high accuracy, decreasing the classification cost, lowering the possibility of tumor progression, and helping the pathologist choose suitable follow-up and treatment plans. Future work will focus on merging handcrafted features used in the literature with DL techniques. Also, further analysis will be conducted on using other DL techniques to analyze childhood MB classes.

## Data Availability Statement

The dataset analyzed for this study can be found in the IEEE Dataport [https://ieee-dataport.org/open-access/childhood-medulloblastoma-microscopic-images].

## Author Contributions

The author confirms being the sole contributor of this work and has approved it for publication.

## Conflict of Interest

The author declares that the research was conducted in the absence of any commercial or financial relationships that could be construed as a potential conflict of interest.
