# Supplementary material for: CoMB-Deep: Composite Deep Learning-Based Pipeline for Classifying Childhood Medulloblastoma and Its Classes
Source: Front Neuroinform. 2021 May 28;15:663592. doi: 10.3389/fninf.2021.663592 (PMC8193683; doi:10.3389/fninf.2021.663592)
Supplement: Supplementary file 1 [file Table_1.docx]

**Table S.1** The names and input/output dimensions of ResNet-50 CNN’s layers

| **Layer Label** | **Input Layer Dimension** | **Output Dimension** |
| --- | --- | --- |
| Input Layer | | 224 × 224 × 3 |
| Conv1 | 112 × 112 × 64 | Filter size = 7 × 7  Number of filters = 64  Stride = 2  Padding = 3 |
| pool1 | 56 × 56 × 64 | Pooling size = 3 × 3  Stride = 2 |
| conv2_x | 56 × 56 × 64 | $\left[ \begin{matrix} 1 \times1. & 64 \\ 3 \times3. & 64 \\ 1 \times1. & 256 \end{matrix} \right]$ $\times3$ |
| conv3_x | 28 × 28 × 128 | $\left[ \begin{matrix} 1 \times1. & 128 \\ 3 \times3. & 128 \\ 1 \times1. & 512 \end{matrix} \right]$ $\times4$ |
| conv4_x | 14 × 14 × 256 | $\left[ \begin{matrix} 1 \times1. & 256 \\ 3 \times3. & 256 \\ 1 \times1. & 1024 \end{matrix} \right]$ $\times6$ |
| conv5_x | 7 × 7 × 512 | $\left[ \begin{matrix} 1 \times1. & 512 \\ 3 \times3. & 512 \\ 1 \times1. & 2048 \end{matrix} \right]$ $\times3$ |
| Average pooling | | Pool size = 7 × 7  Stride = 7 |
|  |  | 1 × 1 |
| FC Layer | | 1000 |
