# Supplementary material for: CoMB-Deep: Composite Deep Learning-Based Pipeline for Classifying Childhood Medulloblastoma and Its Classes
Source: Front Neuroinform. 2021 May 28;15:663592. doi: 10.3389/fninf.2021.663592 (PMC8193683; doi:10.3389/fninf.2021.663592)
Supplement: Supplementary file 2 [file Table_2.docx]

**Table S.2** The names, input size, and patch/stride size of the numerous layers of Inception-V3 CNN

| **Layer Label** | **Patch size / Stride** | **Input Size** |
| --- | --- | --- |
| Conv | 3 x 3 /2 | 229 x 229 x 3 |
| Conv | 3 x 3 /1 | 149 x 149 x 32 |
| Conv padded | 3 x 3 /1 | 147 x 147 x 32 |
| Pool | 3 x 3 /2 | 147 x 147 x 64 |
| Conv | 3 x 3 /1 | 73 x 73 x 64 |
| Conv | 3 x 3 /2 | 71 x 71 x 80 |
| Conv | 3 x 3 /1 | 35 x 35 x 192 |
| 3 x Inception | - | 35 x 35 x 288 |
| 5 x Inception | - | 17 x 17 x 768 |
| 2 x Inception | - | 8 x 8 x 1280 |
| Pool | 8 x 8 | 8 x 8 x 2048 |
| Linear | - | 1 x 1 x 2048 |
| Softmax | - | 1 x 1 x 1000 |
