# Supplementary material for: CoMB-Deep: Composite Deep Learning-Based Pipeline for Classifying Childhood Medulloblastoma and Its Classes
Source: Front Neuroinform. 2021 May 28;15:663592. doi: 10.3389/fninf.2021.663592 (PMC8193683; doi:10.3389/fninf.2021.663592)
Supplement: Supplementary file 3 [file Table_3.docx]

**Table S.3** The names and input/output dimensions of DenseNet-201 CNN’s layers.

| **Layer Label** | **Input Layer Dimension** | **Output Dimension** |
| --- | --- | --- |
| Input Layer | | 224 × 224 × 3 |
| Convolution | 112 × 112 | Filter size = 7 × 7  Stride = 2  Padding = 3 |
| pooling | 56 × 56 | Maximum Pooling = 3 × 3  Stride = 2 |
| Dense Block 1 | 56 × 56 | $\left[ \begin{matrix} 1 & \times1 \\ 3 & \times3 \end{matrix} \right]\times6$ |
| Transition Layer 1 | 56 × 56 | 1 x 1 convolution |
|  | 28 × 28 | 2 x 2 average pooling, stride =2 |
| Dense Block 2 | 28 × 28 | $\left[ \begin{matrix} 1 & \times1 \\ 3 & \times3 \end{matrix} \right]\times12$ |
| Transition Layer 2 | 28 x 28 | 1 x 1 convolution |
|  | 14 x 14 | 2 x 2 average pooling, stride =2 |
| Dense Block 3 | 14 × 14 | $\left[ \begin{matrix} 1 & \times1 \\ 3 & \times3 \end{matrix} \right]\times48$ |
| Transition Layer 3 | 14 x 14 | 1 x 1 convolution |
|  | 7 x 7 | 2 x 2 average pooling, stride =2 |
| Dense Block 4 | 7 x 7 | $\left[ \begin{matrix} 1 & \times1 \\ 3 & \times3 \end{matrix} \right]\times32$ |
| Pooling | | Average Pooling= 7 × 7  Stride = 7 |
|  |  | 1 × 1 |
| FC Layer | | 1000 |
