# Supplementary material for: CoMB-Deep: Composite Deep Learning-Based Pipeline for Classifying Childhood Medulloblastoma and Its Classes
Source: Front Neuroinform. 2021 May 28;15:663592. doi: 10.3389/fninf.2021.663592 (PMC8193683; doi:10.3389/fninf.2021.663592)
Supplement: Supplementary file 4 [file Table_4.docx]

**Table S.4** The names, input size, and filter/stride size of the numerous layers of MobileNet CNN.

| **Layer Label** | **Input Layer Dimension** | **Filter and Stride Size** |
| --- | --- | --- |
| Convolution/S2 | 224 x 224 x 3 | Filter size = 3 x 3 x 3 x 32  Stride = 2 |
| Convolution/dw^8^/S1 | 112 x 112 x 32 | Filter size = 3 × 3 x 32 dw  Stride = 1 |
| Convolution/S1 | 112 x 112 x 32 | Filter size 1 x 1 x 32 x64  Stride = 1 |
| Convolution/dw/S2 | 112 x 112 x 64 | Filter size = 3 × 3 x 64 dw  Stride = 2 |
| Convolution/S1 | 56 x 56 x 64 | Filter size 1 x 1 x 64 x 128  Stride = 1 |
| Convolution/dw/S1 | 56 x 56 x 128 | Filter size = 3 × 3 x 128 dw  Stride = 2 |
| Convolution/S1 | 56 x 56 x 128 | Filter size 1 x 1 x 128 x 128  Stride = 1 |
| Convolution/dw/S2 | 56 x 56 x 128 | Filter size = 3 × 3 x 128 dw  Stride = 2 |
| Convolution/S1 | 28 x 28 x 128 | Filter size 1 x 1 x 128 x 256  Stride = 1 |
| Convolution/dw/S1 | 28 x 28 x 256 | Filter size = 3 × 3 x 256 dw  Stride = 1 |
| Convolution/S1 | 28 x 28 x 256 | Filter size 1 x 1 x 256 x 256  Stride = 1 |
| Convolution/dw/S2 | 56 x 56 x 128 | Filter size = 3 × 3 x 256 dw  Stride = 2 |
| Convolution/S1 | 14 x 14 x 256 | Filter size 1 x 1 x 256 x 512  Stride = 1 |
| 5 x Convolution dw/S1  5 x Convolution S1 | 14 x 14 x 512  14 x 14 x 512 | Filter size = 3 × 3 x 512 dw  Filter size 1 x 1 x 512 x 512  Stride = 1 |
| Convolution dw/S2 | 14 x 14 x 512 | Filter size = 3 × 3 x 512 dw  Stride 2 |
| Convolution/S1 | 7 x 7 x 512 | Filter size 1 x 1 x 512 x 512  Stride = 1 |
| Convolution dw/S2 | 7 x 7 x 1024 | Filter size = 3 × 3 x 1024 dw  Stride 2 |
| Convolution/S1 | 7 x 7 x 1024 | Filter size 1 x 1 x 1024 x 1024  Stride = 1 |
| Pooling | | Average Pooling= 7 × 7  Stride = 1 |
|  |  | 1 × 1 x 1024 |
| FC Layer | | 1 x1 x 1000 |

dw^*^ stands for depthwise
