# Supplementary material for: CoMB-Deep: Composite Deep Learning-Based Pipeline for Classifying Childhood Medulloblastoma and Its Classes
Source: Front Neuroinform. 2021 May 28;15:663592. doi: 10.3389/fninf.2021.663592 (PMC8193683; doi:10.3389/fninf.2021.663592)
Supplement: Supplementary file 5 [file Table_5.docx]

**Table S.5** The names, output size, kernel/stride size , and output channel size of the numerous layers of of ShuffleNet CNN.

| **Layer Label** | **Output Dimension** | **Kernel Size** | **Stride Size** | **Repeat** | **Output Channels (C clusters)** | | | | |
| --- | --- | --- | --- | --- | --- | --- | --- | --- | --- |
|  |  |  |  |  | **C=1** | **C=2** | **C=3** | **C=4** | **C=8** |
| Input Image | 224 × 224 | - |  |  | 3 | 3 | 3 | 3 | 3 |
| Conv1 | 112 x 112 | 3 x 3 | 2 | 1 | 24 | 24 | 24 | 24 | 24 |
| Maximum Pooling | 56 × 56 | 3 x 3 | 2 |  |  |  |  |  |  |
| Stack of Shuffle Elements (Stage 2) | 28 × 28 | - | 2 | 1 | 144 | 200 | 240 | 272 | 384 |
|  | 28 × 28 | - | 1 | 3 | 144 | 200 | 240 | 272 | 384 |
| Stack of Shuffle Elements (Stage 3) | 14 × 14 | - | 2 | 1 | 288 | 400 | 480 | 544 | 768 |
|  | 14 x 14 | - | 1 | 7 | 288 | 400 | 480 | 544 | 768 |
| Stack of Shuffle Elements (Stage 4) | 7 x 7 | - | 2 | 1 | 576 | 800 | 960 | 1088 | 1536 |
|  | 7 x 7 | - | 1 | 3 | 576 | 800 | 960 | 1088 | 1536 |
| Global Pooling | 1 x 1 | 7 x 7 |  |  |  |  |  |  |  |
| Fully Connected | 14 x 14 |  |  |  | 1000 | 1000 | 1000 | 1000 | 1000 |
|  | 7 x 7 |  |  |  |  |  |  |  |  |
