# Supplementary material for: CoMB-Deep: Composite Deep Learning-Based Pipeline for Classifying Childhood Medulloblastoma and Its Classes
Source: Front Neuroinform. 2021 May 28;15:663592. doi: 10.3389/fninf.2021.663592 (PMC8193683; doi:10.3389/fninf.2021.663592)
Supplement: Supplementary file 6 [file Table_6.docx]

**Table S.6** The names, output size, kernel/stride size, depth size, and squeeze and expand sizes of the numerous layers of of SqueezeNet CNN.

| **Layer Label** | **Output Dimension** | **Kernel Size** | **Stride Size** | **Depth** | **Squeeze** | **Expand** | | |
| --- | --- | --- | --- | --- | --- | --- | --- | --- |
|  |  |  |  |  | **S_1x1(#1x1)_** | **C=3** | **C=4** | **C=8** |
| Input Image | 224 × 224 x3 | - |  |  |  |  |  |  |
| Conv1 | 111 x 111 x 3 | 7 x 7(x96) | 2 | 1 |  |  |  |  |
| Maximum Pooling 1 | 55 × 55 x 96 | 3 x 3 | 2 | 0 |  |  |  |  |
| Fire 2 | 55 × 55 x 128 | - | - | 2 | 16 | 64 | 64 |  |
| Fire 3 | 55 × 55 x 128 | - | - | 2 | 16 | 64 | 64 |  |
| Fire 4 | 55 × 55 x 256 | - | - | 2 | 32 | 128 | 128 |  |
| Maximum Pooling 4 | 27 × 27 x 256 | 3 x 3 | 2 | 0 |  |  |  |  |
| Fire 5 | 27 × 27 x 256 | - | - | 2 | 32 | 128 | 128 |  |
| Fire 6 | 27 × 27 x 384 | - | 1 | 2 | 48 | 192 | 192 |  |
| Fire 7 | 27 × 27 x 384 | - |  | 2 | 48 | 192 | 192 |  |
| Fire 8 | 27 × 27 x 512 | - |  | 2 | 64 | 256 | 256 |  |
| Maximum Pooling 8 | 27 × 27 x 512 | 3 x 3 | 2 | 0 |  |  |  |  |
| Fire 9 | 27 × 27 x 512 | - |  | 2 | 64 | 256 | 256 |  |
| Conv 10 | 13 × 13 x 1000 | 1x1 (x1000) | 1 | 1 |  |  |  |  |
| Average Pooling 10 | 1 × 1 x 1000 | 13x 13 | 1 | 0 |  |  |  |  |
