# Supplementary material for: CoMB-Deep: Composite Deep Learning-Based Pipeline for Classifying Childhood Medulloblastoma and Its Classes
Source: Front Neuroinform. 2021 May 28;15:663592. doi: 10.3389/fninf.2021.663592 (PMC8193683; doi:10.3389/fninf.2021.663592)
Supplement: Supplementary file 7 [file Table_7.docx]

**Table S.7** The name, filter size, stride size, and output size of the numerous layers of of DarkNet CNN.

| **Layer Label** | **Filters** | **Size** | **Stride Size** | **Output Size** |
| --- | --- | --- | --- | --- |
| Convolutional | 32 | 3 x 3 |  | 256 x 256 |
| Convolutional | 64 | 3 x 3 | 2 | 128 x 128 |
| Convolutional | 32 | 1 x 1 |  | - |
| Convolutional | 64 | 3 x 3 |  | - |
| Residual |  | - |  | 128 x 128 |
| Convolutional | 128 | 3 x 3 | 2 | 64 x 64 |
| Convolutional | 64 | 1 x 1 |  | - |
| Convolutional | 128 | 3 x 3 |  | 1 |
| Residual |  |  |  | 64 x 64 |
| Convolutional | 256 | 3 x 3 | 2 | 32 x 32 |
| Convolutional | 128 | 3 x 3 |  | 2 |
| Convolutional | 256 |  |  |  |
| Residual |  |  |  | 32 x 32 |
| Convolutional | 512 | 3 x 3 | 2 | 1 |
| Convolutional | 256 | 1 x 1 |  |  |
| Convolutional | 512 | 3 x 3 |  |  |
| Residual |  |  |  | 16 x 16 |
| Convolutional | 1024 | 3 x 3 | 2 | 8 x 8 |
| Convolutional | 512 | 1 x 1 |  |  |
| Convolutional | 1024 | 3 x 3 |  |  |
| Residual |  |  |  | 8 x 8 |
