# Supplementary figures and images for: CoMB-Deep: Composite Deep Learning-Based Pipeline for Classifying Childhood Medulloblastoma and Its Classes
Source: Front Neuroinform. 2021 May 28;15:663592. doi: 10.3389/fninf.2021.663592 (PMC8193683; doi:10.3389/fninf.2021.663592)

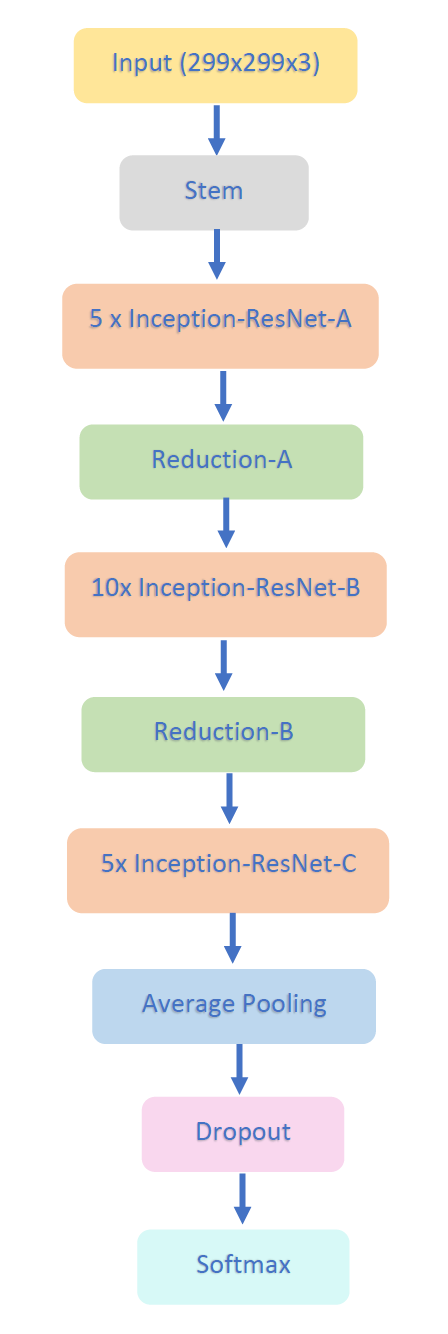

Supplement: Supplementary file 9 [file Image_1.TIF]
